# Mechanical Properties and Environmental Evaluation of Ultra-High-Performance Concrete with Aeolian Sand

**DOI:** 10.3390/ma13143148

**Published:** 2020-07-15

**Authors:** Hongyan Chu, Fengjuan Wang, Liguo Wang, Taotao Feng, Danqian Wang

**Affiliations:** 1 College of Civil Engineering, Nanjing Forestry University, Nanjing 210037, China; 2 Jiangsu Key Laboratory of Construction Materials, School of Materials Science and Engineering, Southeast University, Nanjing 211189, China; fengjuan19921118@sina.com (F.W.); wlg_seu@sina.com (L.W.); taotao_feng@yeah.net (T.F.); 3 Advanced and Innovative Materials (AIM) Group, Department of Civil, Environmental and Geomatic Engineering, University College London, London WC1E 6BT, UK; danqian.wang.16@ucl.ac.uk

**Keywords:** ultra-high-performance concrete, microstructure, Young’s modulus, mechanical properties, aeolian sand, environmental assessment

## Abstract

Ultra-high-performance concrete (UHPC) has received increasing attention in recent years due to its remarkable ductility, durability, and mechanical properties. However, the manufacture of UHPC can cause serious environmental issues. This work addresses the feasibility of using aeolian sand to produce UHPC, and the mix design, environmental impact, and mechanical characterization of UHPC are investigated. We designed the mix proportions of the UHPC according to the modified Andreasen and Andersen particle packing model. We studied the workability, microstructure, porosity, mechanical performance, and environmental impact of UHPC with three different water/binder ratios. The following findings were noted: (1) the compressive strength, flexural strength, and Young’s modulus of the designed UHPC samples were in the ranges of 163.9–207.0 MPa, 18.0–32.2 MPa, and 49.3–58.9 GPa, respectively; (2) the compressive strength, flexural strength, and Young’s modulus of the UHPC increased with a decrease in water/binder ratio and an increase in the steel fibre content; (3) the compressive strength–Young’s modulus correlation of the UHPC could be described by an exponential formula; (4) the environmental impact of UHPC can be improved by decreasing its water/binder ratio. These findings suggest that it is possible to use aeolian sand to manufacture UHPC, and this study promotes the application of aeolian sand for this purpose.

## 1. Introduction

Desertification is one of the most serious global environmental degradation problems, and deserts currently cover approximately 1/3 of the Earth’s land area. In China, the desertification area is approximately 1.54 million km^2^, accounting for approximately 16% of China’s land mass. With the development of the Chinese economy, the construction of China’s infrastructure has undergone sustained and rapid development, with the result that building sand availability cannot meet the demand. In particular, there is an acute shortage of medium and coarse sand in north-western China. In addition, in many regions of the Earth (particularly in large arid areas), the shortage of building sand is also a growing problem. However, as a type of solid waste, aeolian sand is very abundant in these regions. Therefore, the development and utilization of aeolian sand have a practical significance from the perspective of sustainable development and have far-reaching social significance and practical engineering value for these areas.

Studies have been conducted on aeolian sand to evaluate its suitability as a building material. Early research was conducted by Khan [1], who analyzed the chemical and geotechnical properties of aeolian sand collected from the Sahara Desert in Libya, and then systematically discussed various aspects related to expressway construction in arid areas. Detailed experimental investigations were performed by Al-Sanad et al. [2] to obtain the geotechnical properties of dune sand collected from four sites in Kuwait. Yuan et al. [3] and Al-Taie et al. [4] examined the engineering properties of aeolian sand and also investigated the possibility for use as a building material. In addition, other researchers have pointed out that the use of aeolian sand as a fine aggregate in common cement mortar (or concrete) is a practical application [5,6].

Recently, Dong et al. [7] conducted an investigation on the damage evolution in lightweight aggregate concrete containing aeolian sand. The findings suggested that the optimal replacement quantity of river sand with aeolian sand in lightweight-aggregate concrete was 20–30%, and the incorporated aeolian sand had double effects on the damage of this kind of concrete. If the incorporated amount of aeolian sand was higher than 30%, the damage in the concrete was increased. This damage was reduced when the incorporation amount of aeolian sand was less than 30%.

Lopez-Querol et al. [8] investigated the carrying capacity of cement-stabilized aeolian sand under confined and unconfined conditions with the purpose of using this type of sand in the construction of roads or railways. They concluded that the application of aeolian sand in roads or railways was feasible. However, there are few reports at home or abroad concerning the application of aeolian sand in ultra-high-performance concrete (UHPC). In addition, the properties of concrete are sensitive to environmental effects, and thus researchers also investigated other procedures to improve the behavior of the concrete under various environmental conditions [9,10,11].

Tremendous progress has been made in relation to the performance of concrete, due to the development of concrete technology. UHPC is a new-type of cementitious composite that is noted for its high mechanical strength and superior durability. Jiang et al. [12] studied the feasibility of using recycled sand to replace natural sand to produce ultra-high-performance cement-based composites (UHPCCs). They found that the properties of the recycled sand UHPCCs were essentially the same as the UHPCCs incorporating natural sand when the substitution rate of recycled sand was lower than 50%.

However, the production of UHPC is relatively expensive due to the utilization of substantial amounts of binders, supplementary cementitious materials, and steel fibres. In addition, when 1000 kg cement clinker is manufactured, approximately 1000 kg of carbon dioxide (a type of greenhouse gas) is produced. Researchers estimated that the total amount of carbon dioxide produced by cement reaches approximately 5–7% of the global anthropogenic carbon dioxide emissions. Thus, the sustainability of UHPC has recently attracted extensive attention, and more efforts should be made to find sustainable solutions for UHPC production.

Various methods have been presented to improve the environmental impact of UHPC. These methods include decreasing the cement content, utilizing supplementary cementitious materials, optimizing the mixture of UHPC, incorporating recycled materials in UHPC, and increasing the durability of UHPC. However, the environmental impact of UHPC demands reasonable and viable production methods. Damineli et al. [13] used the binder intensity and carbon dioxide intensity to define the ecological efficiency of concrete. The binder intensity index denotes the binder needed for adding 1 MPa compressive strength of concrete, whereas the carbon dioxide intensity index denotes the greenhouse gas emissions resulting from the production of concrete. Accordingly, the evaluation of the environmental impact of UHPC requires further investigation, as its components are complex.

Typically, the production of UHPC requires many binding materials. Rossi [14] developed a type of ultra-high-performance cementitious composite, in which 1050 kg of cement per cubic meter of concrete was used. Ei-Dieb [15] designed an ultra-high strength concrete with approximately 900 kg of cement and 135 kg of silica fume per cubic meter. Compared to normal concrete, the cement content in UHPC is typically between 900 and 1100 kg/m^3^, which is approximately twice the amount used in normal concrete. Although some studies have demonstrated that it is possible to replace the binding materials in UHPC using limestone powder, fine quartz sand, or calcined clay [16,17,18], it remains a challenging task to find a reasonable balance between the binder content and the comprehensive properties of UHPC.

Brouwers and Radix [19] stated that the improved mechanical properties and superior durability of concrete mainly result from the optimum packing of its granular components. However, in most studies, the UHPC mixtures are directly presented with no detailed explanation [20,21,22,23,24,25]. Clearly, the available research regarding the mix proportion design of UHPC with an optimized particle packing is inadequate, and some raw materials are not well used in UHPC. In recent years, researchers attempted to design earth-moist concrete [26], ultra-high-performance fibre-reinforced concrete [27], and UHPC [28,29] according to the particle packing theory. In addition, Wang et al. [30] developed a type of UHPC incorporating recycled coral-based materials supported by the particle packing theory. As mentioned above, most investigations have only focused on the mix design or relative properties of UHPC and ignored the environmental impact.

In this work, we investigated the mix proportion design, mechanical characterization, and environmental impact of UHPC made with aeolian sand. The mix proportions of UHPC were designed according to the modified Andreasen and Andersen particle packing theory. 40 wt.% cement was replaced by fly ash and silica fume in the UHPC designed in this study. Two different steel fibre volume fractions were adopted to assess the influence of the steel fibre content on the mechanical performance and environmental impact of UHPC. The workability, microstructure, porosity, mechanical performance (e.g., compressive strength, flexural strength, and Young’s modulus), and environmental impact of UHPCs with three different water/binder ratios were comprehensively studied. An effort was also made to assess the environmental impact of the designed UHPC by utilization of the environmental impact indexes. The findings of this research will improve the application of aeolian sand in UHPC.

## 2. Materials and Experimental Methods

### 2.1. Raw Materials

**Figure 1:**
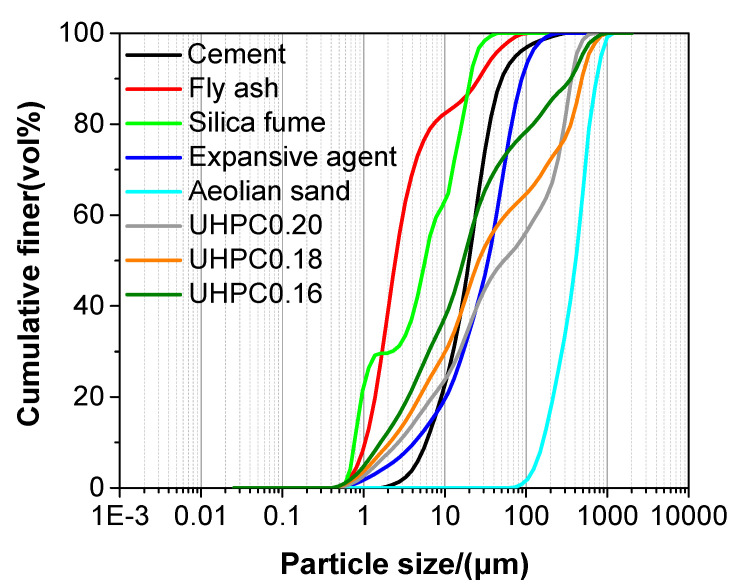
The particle size distribution of the raw materials.

The binder materials used in the study were P∙II 52.5 cement, silica fume, and fly ash. Aeolian sand collected from the Taklimakan desert (Xinjiang, China) was utilized as a fine aggregate. The chemical composition of the cement, fly ash, and aeolian sand are presented in Table 1. The SiO_2_ content in the silica fume was higher than 99.00 wt.%. The specific surface and specific gravity of cement were 362.20 m^2^/kg and 3.15, respectively, and the specific surface and specific gravity of silica fume were 2.79 × 10^4^ m^2^/kg and 2.22, respectively. The particle size distribution of the raw materials is presented in Figure 1.

We utilised a superplasticiser of polycarboxylate to obtain a satisfactory workability of the UHPC, and the water-reducing rate of the superplasticiser was 33.9%. The size of the steel fibres incorporated in the work was 0.2 mm (diameter) × 13 mm (length), and the tensile strength was approximately 3.0 GPa. A CaO expansive agent was utilised in the study, and its content was 5 wt.% of cement.

### 2.2. Mix Proportion Design of UHPC

In the mix proportion design procedure, the UHPC mixtures were adequately designed according to the particle packing theory. The modified Andreasen & Andersen particle packing theory [31] was applied in this work, which is expressed as follows:

(1) $$V\left(S\right)=\left(S^{d}-S_{min}^{d}\right)/\left(S_{max}^{d}-S_{min}^{d}\right)$$

where S is the particle size, and V(S) is the cumulative volume fraction smaller than size S. d, $S_{min}$, and $S_{max}$ are the distribution modulus, minimum particle size, and maximum particle size, respectively.

In this study, d was set at 0.23 based on previous research findings [27,29]. For how to design the UHPC using particle packing theory, the details are available in published papers [27,28,32]. For simplicity, the theoretical grading curves that were calculated from Equation (1) of UHPC with different water/binder ratios are also plotted in Figure 1. According to the findings of Wille and Boisvert-Cotulio [33], the aggregate/cement ratio of the UHPC mixtures was 1.33 (see Table 2).

The mixing proportions of the UHPC were designed in accordance with the optimised particle packing theory, and they are listed in Table 2. In total, three different types of UHPC were designed, and their water/binder ratios were 0.20, 0.18, and 0.16, respectively. For UHPC, the steel fibre content is typically 2–5 vol% [34]. Thus, two different steel fibre contents (2.5 vol% and 3.0 vol%) were used for each water/binder ratio in the study.

### 2.3. Specimen Preparation

To obtain a homogenous mixture, the P∙II 52.5 cement, fly ash, and silica fume were mixed using a mixer for 5 min, then these binders were mixed with fine aggregate for 5 min. Subsequently, the dry materials were mixed with water and the superplasticiser for 10 min, and the steel fibre was added during the 5 min of final mixing. The superplasticiser was mixed evenly with a part of the water, and this mixed solution was then poured into the dry materials.

After mixing, cubic (size: 70.7 mm × 70.7 mm × 70.7 mm) and prismatic (sizes: 70 mm × 70 mm × 280 mm and 70.7 mm × 70.7 mm × 220 mm) specimens of UHPC were cast. First, the surfaces of the samples were covered with plastic sheeting after casting. Secondly, the samples were cured for approximately 48 h under room temperature conditions before demoulding. Thirdly, the samples were cured for 28 days under the condition that the temperature was 21 ± 1 °C and the relative humidity was above 95%. Five cubic, six prismatic (size: 70 mm × 70 mm × 280 mm), and ten prismatic (size: 70.7 mm × 70.7 mm × 220 mm) specimens were made for each mixture of UHPC. All the specimens of UHPC were prepared according to the Chinese standard GB 50204-2015 [35].

### 2.4. Testing Methodology

The slump flow of the UHPC samples was measured with a normal concrete slump cone set based on the Chinese standard GB 50119-2013 [36]. (similar to ASTM C143). In this study, the microstructure of the UHPC was characterized using 3D environmental scanning electronic microscopy (ESEM). The specimens were crushed into small fragments and immersed in alcohol to prevent their further hydration when the curing treatment was finished. The specimens were then dried and stored in a sealed jar before testing. The porosity and pore size distributions of the UHPC were tested via the mercury intrusion porosimeter (AutoPore IV; Micromeritics, Norcross, GA, USA). The compressive strength of the UHPC was measured via a universal testing machine with a loading rate of 0.80 MPa/s. A four-point bending loading configuration was utilised to test the flexural strength of the UHPC, and the space between the loading points was 210 mm, in which region there was no shear force. The Young’s modulus of the UHPC was measured using a universal testing machine equipped with a micro-deformation testing facility, and the loading rate of the experiment was also 0.8 MPa/s.

All the mechanical properties of the UHPC were measured after the curing treatment based on the Chinese standard GB/T 17617-1999 [37]. (which is similar to ASTM C109). The UHPC specimen size for compressive strength was 70.7 mm × 70.7 mm × 70.7 mm, and the specimen sizes for the four-point bending flexural strength test and Young’s modulus test were 70 mm × 70 mm × 280 mm and 70.7 mm × 70.7 mm × 220 mm, respectively. The porosity, compressive strength, and flexural strength of the UHPC were measured three repeated times, and the Young’s modulus of the UHPC was tested six repeated times.

### 2.5. Environmental Assessment

In this work, the embedded carbon dioxide index (ECI) and the unit cement compressive strength contribution index (UCI) were used to characterize the environmental impact of the UHPC. The definitions of these two indexes are as follows:

(2) $$\;\mathrm{ECI}=\mathrm{embodied}\;\mathrm{carbon}\;\mathrm{dioxide}\;\left(\mathrm{kg}/m^{3}\right)/\mathrm{compressive}\;\mathrm{stress}\;\left(\mathrm{MPa}\right)$$

(3) $$\mathrm{UCI}=\mathrm{compressive}\;\mathrm{stress}\;\left(\mathrm{MPa}\right)/\;\mathrm{cement}\;\mathrm{content}\;\left(\mathrm{kg}/m^{3}\right).$$

According to the findings of Long et al. [38], the embedded carbon dioxide of UHPC was calculated during the phases of processing raw materials and the curing of concrete. Hence, the carbon dioxide emissions of UHPC is the sum of the embedded carbon dioxide in its raw materials and curing process. The embedded carbon dioxide of the raw materials used in UHPC production has been studied by many researchers and, according to these previous studies, the embedded carbon dioxide of each type of raw material is presented in Table 3. The embedded carbon dioxide of the aeolian sand is the same as that of the river sand, and the embedded carbon dioxide of the expansive agent is the average embedded carbon dioxide of the air entraining and retarder agents shown in the paper of Long et al. [38]. To ensure the required strength, the specimens of UHPC were cured in a standard curing room for 28 days. The total carbon dioxide emissions were calculated from the power consumption of the curing room, namely, 1.19 kw × 24 h × 28 days × 0.997 kg/(kw·h) = 797.2810 kg/m^3^; thus, the average embedded carbon dioxide for each type of UHPC in this study was 132.8802 kg/m^3^.

## 3. Results and Discussion

### 3.1. Workability of UHPC

The slump test results of the UHPCs are presented in Table 4. As shown in Table 4, the slump flow of UHPC decreased with decreasing water/binder ratio, which is in line with the results of UHPC made of river sand reported in the literature [40]. The minimum slump flow of UHPC was 570 mm, which still meets the requirements of self-consolidating concrete. When the steel fibre content was 2.5 vol%, the slump flows of UHPC with water/binder ratios of 0.18 and 0.16 decreased by 13.9% and 30.0%, respectively, compared to the UHPC whose water/binder ratio was 0.20.

When the water/binder ratio remained constant, the slump of UHPC decreased with the rise in steel fibre content, which is consistent with the findings of Alsalman et al. [35], who also found that the workability of UHPC made of river sand was decreased due to the increase of steel fibre. When the steel fibre content increased from 2.5 vol% to 3.0 vol%, the slump flows of UHPCs with water/binder ratios of 0.20, 0.18, and 0.16, decreased by 6.67%, 9.68%, and 9.52%, respectively. On the one hand, the specific surface area was enlarged due to the increment in steel fibre, and thus, the cohesive force between the steel fibre and cement paste was higher [41]. On the other hand, the inter-connection of steel fibres in the cement paste generated a skeleton, which could further inhibit the fluidity of the UHPC [28,42].

### 3.2. Microstructure

**Figure 2:**
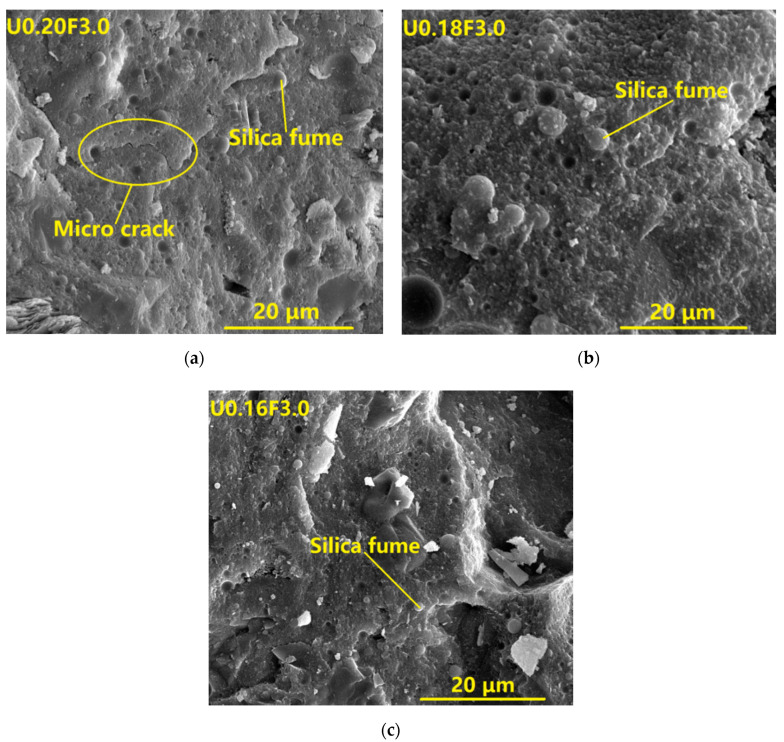
Micrographs of the different kinds of UHPC at 28 days.

The ESEM images of the UHPC samples are presented in Figure 2.

The matrices of U0.18F3.0 and U0.16F3.0 exhibited a continuous microstructure without micro cracks (see Figure 2), and this result was consistent with sacrificial concrete at ambient temperature [43] and UHPC [24]. The matrix of U0.20F3.0 displayed a continuous microstructure with some micro cracks, which was in line with the findings of Meng and Khayat [44]. There was a large quantity of spherical silica fume particles in the matrices of UHPC due to the addition of silica fume, as is vividly depicted in the micrographs of U0.20F3.0, U0.18F3.0, and U0.16F3.0. In addition, the matrices of U0.18F3.0 and U0.16F3.0 were more compact than that of U0.20F3.0, which indicated that the microstructure of UHPC could be improved if the water/binder ratio decreased.

### 3.3. Porosity

**Figure 3:**
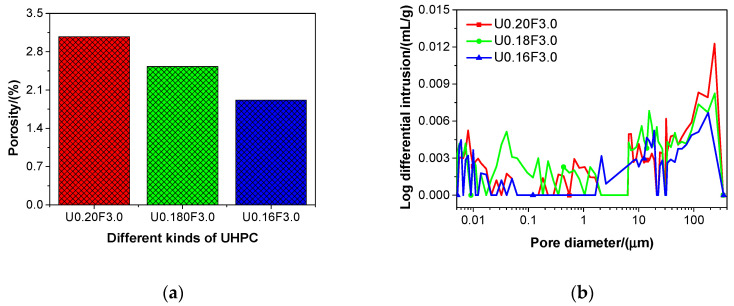
The porosity (**a**) and pore size distribution (**b**) of different kinds of UHPC at 28 days.

Figure 3 presents the porosity and pore size distribution of different types of UHPC. After the curing treatment, the porosities of U0.20F3.0, U0.18F3.0, and U0.16F3.0 were 3.07%, 2.53%, and 1.92%, respectively, which followed the sequence U0.20F3.0 > U0.18F3.0 > U0.16F3.0, as shown in Figure 3a. The porosity of UHPC was lower than that of high-strength concrete [45], and this was similar to the findings of Yu et al. [27], Wang et al. [46], and Jiang et al. [47]. The porosities of U0.18F3.0 and U0.16F3.0 were invariably lower than that of U0.20F3.0, which suggested that the porosity of UHPC diminished when the water/binder ratio of UHPC was reduced. The air content of UHPC increased with the decreasing water/binder ratio; thus, the porosity might increase again when the newly generated hydration products were not sufficient to fill the pores resulting from the air entrained in the fresh UHPC.

With respect to the pore size distribution of U0.20F3.0, U0.18F3.0, and U0.16F3.0, there were multi peaks in the different types of UHPC, as presented in Figure 3b. The changing trend of the pore diameter in UHPC was in agreement with that in sacrificial concrete at 25 °C [48]. In addition, the changing trends of pore diameter in different kinds of UHPC were fairly close to each other. In general, the magnitude of the Log differential intrusion for different types of UHPC followed the sequence U0.20F3.0 > U0.18F3.0 > U0.16F3.0, which was in accordance with the porosity results of the UHPC.

### 3.4. Compressive Strength

**Figure 4:**
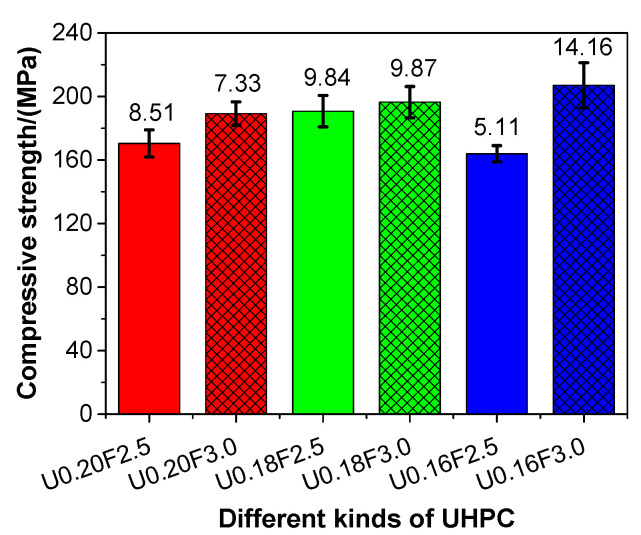
The compressive strength of different kinds of UHPC at 28 days.

Figure 4 presents the compressive strength of different types of UHPC with aeolian sand. As the water/binder ratio declined, the compressive strength of UHPC gradually increased (except for U0.16F2.5), as shown in the figure. These results were different (in part) from those presented by Yu et al. [49]. The compressive strength of UHPC produced in this work was in the range of 163.9–207.0 MPa, which is clearly higher than the requirement for the mechanical strength of UHPC (i.e., compressive strength > 150 MPa) [50]. In general, the compressive strength of UHPC increased as the porosity reduced (Figure 3a), and this result was in line with the findings of Siddique [51].

Regarding the impact of the steel fibre content on the compressive strength of UHPC, this was clearly increased when the steel fibre content increased from 2.5 to 3.0 vol%, which suggested that the adoption of steel fibre was conducive to the improvement in the compressive strength of UHPC. This result is line with the finding of Su et al. [52], who also found that the compressive strength of UHPC made of natural sand was increased due the increase of steel fibre. When the water/binder ratios of UHPC with aeolian sand were 0.20, 0.18, and 0.16, the compressive strength of UHPC increased by 11.03%, 2.99%, and 26.30%, respectively, with the increasing steel fibre content. In most instances, the compressive strength of the concrete declined gradually as the water content was increased, as the excessive water could increase the porosity of concrete. However, a large amount of cement and supplementary cementitious materials, and limited water, were used in the UHPC. A large amount of water was absorbed by the powders and could not react with the cement when the water/binder ratio was small, which, in turn, reduced the compressive strength of the UHPC. This was possibly the reason why the compressive strength of U0.16F2.5 was the lowest.

### 3.5. Flexural Strength

**Figure 5:**
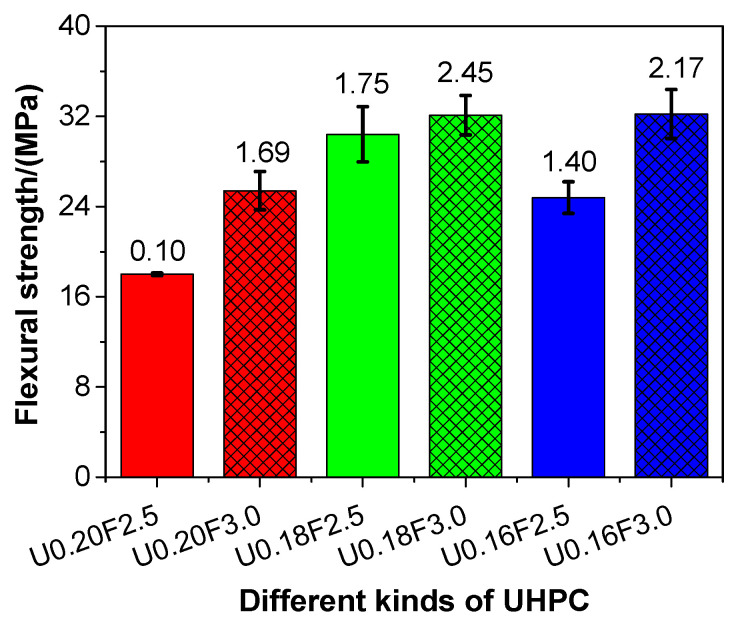
The flexural strength of different kinds of UHPC at 28 days.

Figure 5 presents the flexural strength of UHPCs with different water/binder ratios. As shown in Figure 5, the flexural strength of UHPC slowly increased, except for U0.16F2.5, with the decrease in the water/binder ratio. The flexural strength of the UHPC ranged from 18.0 to 32.2 MPa. The changing trends of flexural strength of the UHPC were in line with those of the compressive strength of UHPC. In addition, with the decrease in porosity (Figure 3a), the flexural strength of UHPC increased in this study, and this result was in line with the findings reported in the literature [50]. The flexural strength of the UHPC increased when the steel fibre content grew from 2.5 to 3.0 vol%, which indicated that the flexural strength of the UHPC could be improved by adopting steel fibres.

This result is consistent with the previous literature [52], which suggested that the flexural strength of UHPC produced by natural sand increased with the increase of steel fibre. Specifically, when the water/binder ratios of UHPC were 0.20, 0.18, and 0.16, the flexural strength of the UHPC increased by 41.11%, 5.59%, and 29.84%, respectively, due to the increasing steel fibre content. The increase in magnitude of the flexural strength was higher than that of the compressive strength of UHPC. Thus, the flexural strength of UHPC could be more easily improved than its compressive strength with the addition of steel fibres.

**Figure 6:**
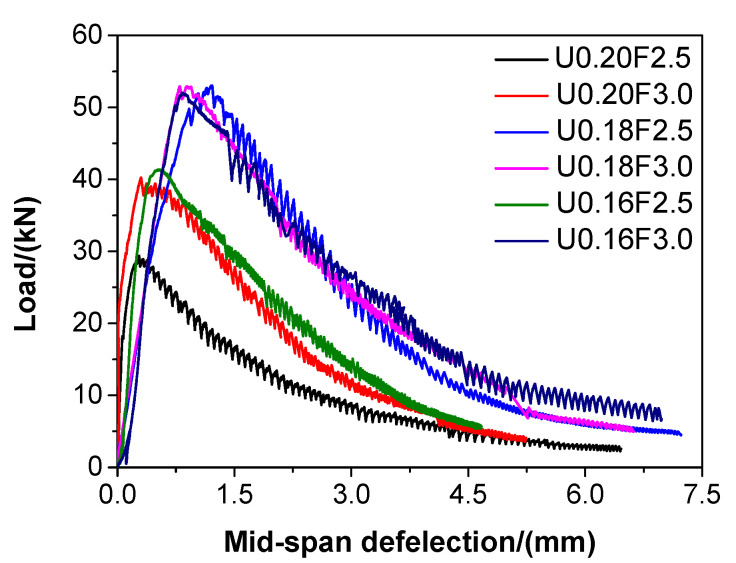
Load-deflection relationships of the UHPCs.

The load-deflection curves of different types of UHPC are illustrated in Figure 6. The load of UHPC increased linearly with the increase of mid-span deflection before reaching the maximum, and then degraded in a plastic manner due to the incorporation of steel fibres.

According to Bazant [53], the fracture energy of concrete materials denotes the needed energy for crack propagation in these kinds of materials. The fracture energy is obtained via Equations as follows:

(4) $$P_{0}=4M_{0}/l$$

(5) $$W_{F}=W_{0}+2P_{0}d_{0}$$

where $d_{0}$ is the maximum mid-span displacement, l is the distance between two supports, $M_{0}$ is the moment caused by the self-weight of the specimen, and $W_{0}$ is the work done by a load that is applied, namely, the area enclosed by the load–deflection curve.

Hence, the fracture energy of UHPC was calculated by the following equation:

(6) $$G_{F}=W_{F}/S$$

where $G_{F}$ is the fracture energy and S is the cross-sectional area.

According to Equations (4)–(6), the fracture energies of UHPC could be determined, and they are listed in Table 5. With the decreasing of water/binder ratio, the fracture energy of UHPC gradually increased, except for U0.16F2.5, which suggested that the plasticity of UHPC could be improved if the water/binder ratio of UHPC was decreased. The changing trend of the fracture energy of UHPC was consistent with that of the flexural strength (Figure 5). The fracture energy of UHPC increased when the added steel fibre increased from 2.5 to 3.0 vol%, which indicated that the plasticity of UHPC could be enhanced by adding steel fibres. This result is accordant with Su et al. [52], who suggested that the fracture energy of UHPC produced by natural sand was increased due the increase of steel fibres. In particular, when the water/binder ratios of UHPC were 0.20, 0.18, and 0.16, the fracture energy of UHPC increased by 49.42%, 0.90%, and 43.68%, respectively, owing to the increasing steel fibre content. It can be seen from Figure 6 that the load–deflection curves of U0.18F2.5 and U0.18F3.0 were very close, which indicated that the difference for the fracture energy of these two mixtures was narrow. However, the load-deflection curves of U0.16F2.5 and U0.18F2.5, and U0.16F2.5 and U0.18F3.0, respectively, had dramatic differences, which suggested the difference for the fracture energy of these mixtures were significant. These might be the reasons why the increasing magnitude of fracture energy for different UHPCs was significant. 

### 3.6. Young’s Modulus

**Figure 7:**
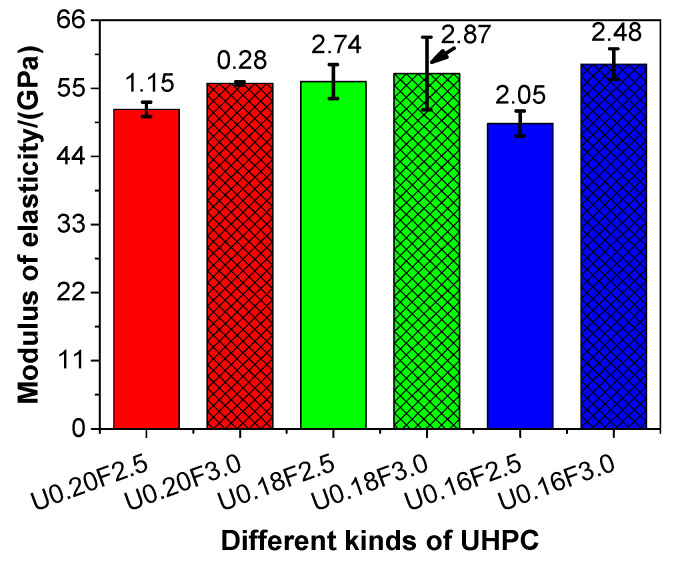
The Young’s modulus of different kinds of UHPC at 28 days.

The Young’s modulus values of the UHPCs are presented in Figure 7.

As shown in Figure 7, the Young’s modulus of the UHPC increased as the water/binder ratio reduced, except for U0.16F2.5. These results suggested that the Young’s modulus of UHPC could be improved with a decrease in the water/binder ratio. The Young’s modulus of the UHPC was in the range of 49.3–58.9 GPa, and these values were higher than those reported in the literature [41]. The stiffness of the UHPC mainly resulted from the sand skeleton [54], as the skeleton generated by the sand particles had a higher stiffness than that of the hardened cement matrix. The Young’s modulus of the UHPC increased when the steel fibre content increased from 2.5 to 3.0 vol%, which indicated that the adoption of steel fibre contributed to enhancing the Young’s modulus of the UHPC. Specifically, the Young’s modulus of the UHPC increased by 8.14%, 2.32%, and 19.47% owing to the increasing steel fibre content when the water/binder ratios were 0.20, 0.18, and 0.16, respectively. These results were in line with the experimental results of Alsalman et al. [41]. In addition, the changing trend of the Young’s modulus of the UHPC was consistent with that of the compressive strength, which revealed that the Young’s modulus of the UHPC might be characterised by its compressive strength. As mentioned above, the mechanical properties (compressive strength, flexural strength, and Young’s modulus) of UHPC increased when the steel fibre content increased; thus, the recommended value of steel fibre was 3.0 vol% in terms of mechanical properties of UHPC.

### 3.7. Compressive Strength—Young’s Modulus Correlation

**Figure 8:**
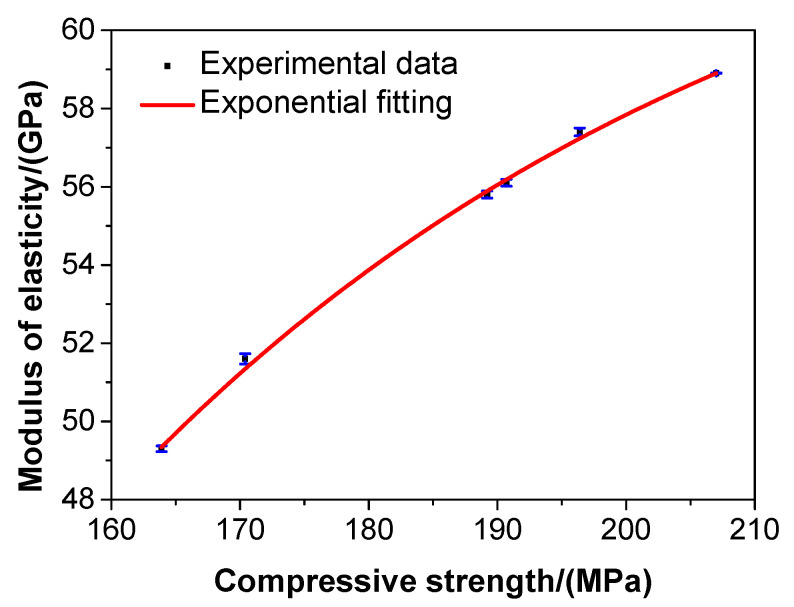
The relationship between the compressive strength and Young’s modulus of the UHPC.

Figure 8 shows the compressive strength—Young’s modulus correlation of the UHPC.

As presented in Figure 8, the Young’s modulus of the UHPC increased gradually with the increase of compressive strength. The compressive strength of the UHPC had a strong correlation with the Young’s modulus, and the compressive strength—Young’s modulus correlation of the UHPC was in an exponential form, shown as the full curve in Figure 8. This exponential form is expressed by the following formula:

(7) $$E=65.88472-429.48946\times e^{-0.01989f_{c}}$$

where E is the Young’s modulus of the UHPC in GPa, and $f_{c}$ is the compressive strength of the UHPC in MPa.

The *R*-square of the above equation was 0.9975, which suggested that the results of data fitting were rather reliable, and that Equation (5) was feasible to evaluate the Young’s modulus of the UHPC in terms of the compressive strength. The empirical formula obtained in the study may not precisely capture the behavior of UHPC with different aggregate types or particle sizes. However, a new empirical equation was developed in the study. In practice, this was a convenient method to assess the Young’s modulus of the UHPC in terms of its compressive strength via the abovementioned equation.

### 3.8. Environmental Assessment 

**Figure 9:**
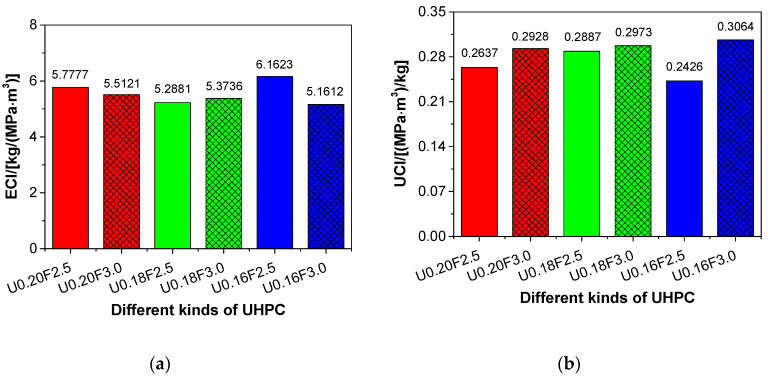
The environmental assessment of UHPC: (**a**) ECI and (**b**) UCI.

The results of ECI and UCI for different types of UHPC are presented in Figure 9. The ECI of UHPC declined with the reduction in water/binder ratio, except for U0.16F2.5, under the same steel fibre content (see Figure 9a), which suggested that the environmental impact of UHPC could be improved with a decrease in the water/binder ratio. When the water/binder ratio decreased, the binder content of UHPC increased, which resulted in the increase of CO_2_ emissions and the compressive strength of UHPC. However, the increase in magnitude of the CO_2_ emissions was lower than that of compressive strength. Therefore, the ECI of UHPC was reduced with the decrease of water/binder ratio, according to Equation (2).

The results of ECI and UCI for different types of UHPC are presented in Figure 9. The ECI of UHPC declined with the reduction in water/binder ratio, except for U0.16F2.5, under the same steel fibre content (see Figure 9a), which suggested that the environmental impact of UHPC could be improved with a decrease in the water/binder ratio. When the water/binder ratio decreased, the binder content of UHPC increased, which resulted in the increase of CO_2_ emissions and the compressive strength of UHPC. However, the increase in magnitude of the CO_2_ emissions was lower than that of compressive strength. Therefore, the ECI of UHPC was reduced with the decrease of water/binder ratio, according to Equation (2).

Contrary to the ECI, the UCI of UHPC increased with the decrease in water/binder ratio (except for U0.16F2.5) under the same steel fibre content (see Figure 9b), which indicated that the environmental impact of UHPC could also be enhanced owing to a decrease in the water/binder ratio. The water/binder ratio of the UHPC declined when the binder content of the UHPC increased, which in turn caused an increase in the compressive strength. The increase in magnitude of the compressive strength was higher than that of the binder content of the UHPC. Hence, the UCI of the UHPC was increased with the decrease in water/binder ratio, according to Equation (3). Overall, the environmental impact of UHPC could be improved by decreasing its water/binder ratio. In addition, the UCI of UHPC increased with the increase of steel fibre content under the same water/binder ratio (see Figure 9b), which suggested that the environmental impact of UHPC could also be enhanced by adding more steel fibres. Therefore, the recommended value of steel fibre was 3.0 vol% in terms of the environmental impact of UHPC. Compared to all other mixtures, the ECI of U0.16F3.0 was the lowest and its UCI was the largest, which suggested that the mix design of U0.16F3.0 had the lowest environmental impact.

**Figure 10:**
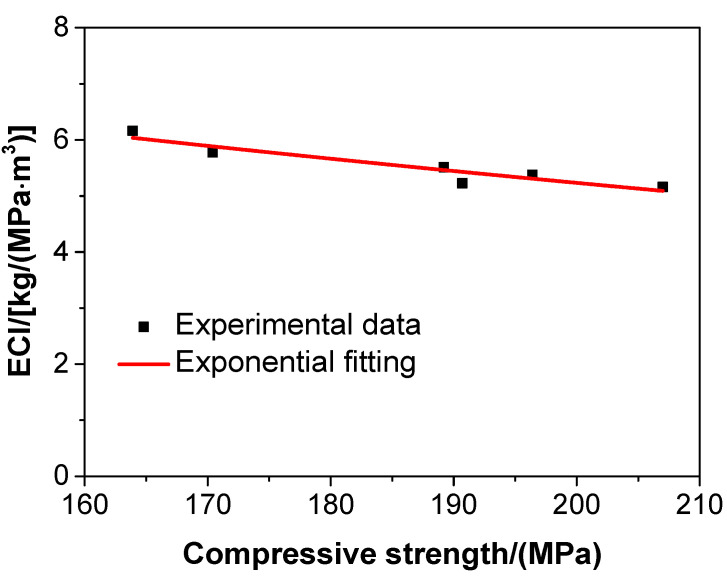
The relationship between the compressive and embedded CO_2_ index of the UHPC.

The compressive strength–ECI correlation of UHPC is shown in Figure 10, where it can be observed that the ECI gradually decreased with the increase in compressive strength in any conditions of the steel fibre content and water/binder ratio of the UHPC in this work. These results are consistent with those found by Shi et al. [55] and Long et al. [38] and in line with the similar results obtained for UHPC and self-compacting concrete. The compressive strength–ECI correlation of UHPC is expressed by the following exponential function:

(8) $$\mathrm{ECI}=11.55178\times e^{-0.00396f_{c}}$$

where ECI is the embedded CO_2_ index of UHPC, and $f_{c}$ is the compressive strength of UHPC in MPa.

The *R*-square of the above equation was 0.8610, which suggested that the exponential fitting was reliable. The compressive strength-ECI correlation of the UHPC displayed a significant negative correlation, which suggested that the environmental impact of UHPC was improved by increasing its compressive strength. In other words, it was feasible to produce UHPC with a lower environmental impact via improving its compressive strength.

In summary, the UHPC mix proportions used in this study were adequately designed according to the particle packing theory, and the experimental investigation provided basic information for the feasibility of aeolian sand in the production of UHPC. The findings indicated that aeolian sand could be successfully applied to develop a UHPC with better mechanical properties than those of the corresponding typical UHPC. That is, it is possible to use aeolian sand to manufacture UHPC. The environmental impact of UHPC could be improved by decreasing its water/binder ratio. As for the mechanism of high mechanical strength of UHPC with aeolian sand, further research is needed.

## 4. Conclusions

This work examined the feasibility of manufacturing UHPC with aeolian sand. The mix proportions of UHPC were designed according to the modified Andreasen & Andersen particle packing theory. Both the ECI and UCI indexes were used to access the environmental impact of UHPC. The major conclusions of the present work are as follows:

(1) The mix proportions of UHPC with aeolian sand were adequately designed according to the particle packing theory, and it is possible to use aeolian sand to manufacture UHPC.
(2) The slump flow of UHPC decreased with a reduction in the water/binder ratio or an increase in the steel fibre content.
(3) The porosities of U0.18F3.0 and U0.16F3.0 were invariably lower than that of U0.20F3.0, which suggests that the porosity of UHPC decreases if the water/binder ratio is reduced.
(4) The compressive strength, flexural strength, and Young’s modulus of UHPC samples with aeolian sand were in the ranges of 163.9–207.0 MPa, 18.0–32.2 MPa, and 49.3–58.9 GPa, respectively.
(5) The compressive strength, flexural strength, and Young’s modulus of UHPC increased with a decrease in the water/binder ratio, except for U0.16F2.5. This indicates that the compressive strength, flexural strength, and Young’s modulus of UHPC are improved when the water/binder ratio is reduced.
(6) The compressive strength, flexural strength, fracture energy, and Young’s modulus of UHPC raised when the steel fibre content increased from 2.5 to 3.0 vol%. This demonstrated that the adoption of steel fibre contributes to enhancing the compressive strength, flexural strength, plasticity, and Young’s modulus of UHPC.
(7) The compressive strength—Young’s modulus correlation of UHPC can be described by an exponential formula. This is a convenient practical method of assessing the Young’s modulus of UHPC in terms of its compressive strength via the empirical formula.
(8) The environmental impact of UHPC was improved by decreasing the water/binder ratio, and the compressive strength–ECI correlation of UHPC displayed a significant negative correlation.
(9) The recommended value of steel fibre was 3.0 vol%, considering the mechanical properties and the environmental impact of UHPC produced in the paper.

## Figures and Tables

**Table 1:** Chemical compositions of the cement, fly ash, and aeolian sand (wt.%).

Materials	Cement	Fly Ash	Aeolian Sand
CaO	64.70	8.38	2.07
SiO_2_	20.40	47.96	81.72
Al_2_O_3_	4.70	30.46	8.19
Fe_2_O_3_	3.38	5.91	1.49
MgO	0.87	2.60	0.84
SO_3_	1.88	1.32	0.08
K_2_O	0.83	1.61	2.12
Na_2_O	-	1.76	1.67
Loss	3.24	-	1.82

**Table 2:** Mixtures of ultra-high-performance concrete (UHPC) (kg/m^3^).

Mixture	U0.20F2.5	U0.20F3.0	U0.18F2.5	U0.18F3.0	U0.16F2.5	U0.16F3.0
Cement	646.2	646.2	660.6	660.6	675.6	675.6
Fly ash	323.1	323.1	330.3	330.3	337.8	337.8
Silica fume	107.7	107.7	110.1	110.1	112.6	112.6
Aeolian sand	861.8	861.8	880.8	880.8	900.6	900.6
Water	215.4	215.4	198.2	198.2	180.2	180.2
Superplasticizer	21.5	21.5	22.0	22.0	22.5	22.5
Expensive agent	32.3	32.3	33.0	33.0	33.8	33.8
Steel fibre	195.0	234.0	195.0	234.0	195.0	234.0
Aggregate/cement ratio (in weight)	1.33	1.33	1.33	1.33	1.33	1.33

**Table 3:** Embedded CO_2_ of the raw materials of ultra-high-performance concrete (UHPC).

Items	Unit	Embedded CO_2_ (kg)	Reference
Cement	kg	0.8324	[39]
Fly ash	kg	0.0090	[38]
Silica fume	kg	0	[39]
Aeolian sand	kg	0.0010	
Water	kg	0.0003	[38]
Superplasticizer	kg	0.7200	[38]
Expensive agent	kg	0.0810	
Steel fibre	kg	1.4965	[39]

**Table 4:** Slump flow of the UHPC samples (mm).

Mixture	U0.20F2.5	U0.20F3.0	U0.18F2.5	U0.18F3.0	U0.16F2.5	U0.16F3.0
Slump flow	900	840	775	700	630	570

**Table 5:** The fracture energy of the UHPC samples (J/m^2^).

Mixture	U0.20F2.5	U0.20F3.0	U0.18F2.5	U0.18F3.0	U0.16F2.5	U0.16F3.0
Fracture energy	60.3	90.1	132.6	133.8	94.1	135.2
